# Identity-Related Factors Protect Well-Being Against Stigma for Transgender and Gender Non-Conforming People

**DOI:** 10.1007/s10508-021-02029-1

**Published:** 2021-10-06

**Authors:** David Matthew Doyle, Christopher T. Begeny, Manuela Barreto, Thomas A. Morton

**Affiliations:** 1 Washington Singer Laboratories, Department of Psychology, University of Exeter, Perry Road, Exeter, Devon, EX4 4QG UK; 2 Lisbon University Institute, Lisbon, Portugal; 3 Department of Psychology, University of Copenhagen, Copenhagen, Denmark

**Keywords:** Transgender, Well-being, Discrimination, Identity affirmation, Self-concept

## Abstract

Relatively little is known about identity-related resilience factors associated with well-being among transgender and gender non-conforming (TGNC) people. Drawing upon theory on stigma-related stress and resilience and work examining group identification as a buffer against discrimination, the aim of the current study was to model perceived discrimination, transgender identification, and gender identity affirmation as predictors of well-being for TGNC people. We also tested whether the positive association between gender identity affirmation and well-being might be explained by the benefits affirmation has for individual self-concept clarity. Participants were 105 TGNC individuals (42% transgender male, 39% transgender female, 19% other gender non-conforming [e.g., non-binary]) recruited through online forums and support groups in the UK and North America who completed an online survey including self-report measures of key constructs. Results from structural equation models demonstrated that: (1) experiences of discrimination were associated with lower well-being overall, but having a stronger transgender identity moderated this association; (2) after adjustment for discrimination and transgender identification, experiences of gender identity affirmation were independently associated with greater well-being for TGNC people. Secondary analyses demonstrated that gender identity affirmation was linked to well-being through reinforcing a strong, internalized sense of clarity about individual self-concept. Results are discussed in terms of the implications for TGNC health and well-being, particularly with regard to the need for supportive, identity-affirming social environments.

## Introduction

Despite rapidly increasing numbers of people identifying as transgender and gender non-conforming (TGNC) across the UK (as well as other European and North American countries; Bouman et al., 2016; Zucker, 2017), relatively little is known about identity-related resilience factors affecting well-being in these populations (Riggle et al., 2011; Testa et al., 2015). Past research has documented considerable disparities between TGNC and cisgender people across various health and well-being outcomes (Feldman et al., 2016; Reisner et al., 2016a). Borrowing from the literature on sexual minority health (Meyer, 2003), these disparities have been explained with reference to the stress engendered by social stigma (Bockting et al., 2016; Hendricks & Testa, 2012; Testa et al., 2015).

To date, however, few studies have investigated potential resilience factors that might protect transgender individuals from the deleterious effects of stigma-related stressors (e.g., exposure to prejudice and discrimination). Along these lines, the focus of the current study was not only those factors that might adversely affect TGNC individuals’ well-being (e.g., perceived discrimination), but also those factors that might help to bolster well-being. In particular, we focus on individuals’ sense of connection and identification with the TGNC community, as well as their experiences of gender identity affirmation (i.e., recognition and verification of one’s chosen gender identity).

### Transgender and Gender Non-Conforming Health and Well-Being

Recent reviews have highlighted many disparities in mental and physical health between TGNC and cisgender people (Feldman et al., 2016; Reisner et al., 2016a). For example, TGNC people suffer from increased rates of psychological distress and depression (e.g., Reisner et al., 2015; Veale et al., 2017), as well as poorer general physical and cardiovascular health (e.g., Meyer et al., 2017; Rider et al., 2018). Of particular note, TGNC people have dramatically elevated rates of attempted suicide (e.g., Reisner et al., 2014, 2015; Veale et al., 2017); for example, 41% of respondents to the National Transgender Discrimination Survey in the U.S. reported a history of attempted suicide (compared to approximately 1.6% of the general U.S. population; Su et al., 2016). Some of these health disparities persist even when comparing gender minorities to sexual minorities, a group that can also be exposed to the stress of social stigma (Su et al., 2016).

Past research has focused largely on mental (ill) health among TGNC populations (and to a lesser extent physical health). Relatively few studies have examined more positive forms of well-being. Well-being refers to “optimal psychological functioning and experience” (Ryan & Deci, 2001) and can be operationalized through a variety of related constructs, including self-esteem and life satisfaction. Consistent with the World Health Organization (1946), we emphasize the fact that health should not be defined simply as the absence of illness and disease (as has been done in much past work on the health of TGNC people), but rather understood more holistically. Studying well-being, rather than just distress and illness, among TGNC people is particularly important given a recent shift toward the depathologization of transgender identities (reflected in the recent removal of “gender identity disorders” from the mental health chapter of the International Classification of Diseases and Related Health Problems Version 11; Reed et al., 2016).

Although research on the well-being of TGNC people is limited, the evidence that does exist suggests poorer outcomes for TGNC people relative to those who are cisgender (Bockting et al., 2016; Stewart et al., 2018). These disparities, as with disparities in mental and physical health, are likely due at least in part to the stress of social stigma. With risk factors related to prejudice and discrimination abundantly evident, it is now essential to investigate resilience factors (including those related to transgender identities) that might protect from the deleterious effects of stigma and bolster health and well-being for TGNC people.

### Transgender Identification

 The term transgender was first used to denote a political and social group identity only in the early 1990’s, drawing together those who defied social norms of gendered embodiment (Stryker, 2006). Because individuals derive self-worth from the groups to which they belong (Tajfel & Turner, 1986), including devalued groups (Branscombe et al., 1999a, 1999b; Doyle & Molix, 2014; Molix & Bettencourt, 2010), self-identification as transgender may be an important source of resilience for TGNC people. Indeed, some models that have adapted the minority stress framework for TGNC populations (e.g., Hendricks & Testa, 2012; Testa et al., 2015) have included transgender identification as a potential protective factor. In line with this theorizing, one past study (Bockting et al., 2013) found that transgender identity pride correlated positively with mental health. By contrast, however, another study found that transgender identity importance was associated with increased depressive symptomatology (McLemore, 2018). Therefore, the direct association between transgender identification and well-being is not entirely evident at this point.

The function of transgender identity in supporting well-being may not be straightforward and direct. Instead identification might moderate responses to experiences of discrimination, and the interplay between these two constructs might flow into feelings of well-being. Indeed, research on other devalued identities (primarily racial/ethnic minority identities) demonstrated that group identification can attenuate associations between discrimination and impaired health and well-being (e.g., Hansen & Sassenberg, 2006; Mossakowski, 2003), thereby buffering individuals against otherwise negative experiences. This is also consistent with how transgender identification has been conceptualized in past work on stigma and health (e.g., Bockting et al., 2013; McLemore, 2018).

### Gender Identity Affirmation

While self-identification is an important aspect of social identity, both internal and external processes shape the way in which people are categorized and subsequently come to see themselves (Barreto & Ellemers, 2003). A threat to the self may arise when one’s internal identity is in conflict with how one is perceived by others (i.e., categorization threat; Branscombe et al., 1999a, 1999b). Conversely, having one’s internal identity affirmed by others may be a positive experience that verifies the self and consequently builds a sense of coherence and self-esteem (Swann, 1983).

Issues of identity affirmation are especially important for TGNC people (McLemore, 2015; Sevelius, 2013) who are often in the process of bringing their external self-presentation closer to how they feel inside, what is designated as gender identity transition. In this process, affirming social environments (including in interactions with friends and family, as well as healthcare practitioners and the general public) can be critical to support health and well-being (Reisner et al., 2016a, 2016b, 2016c; Sevelius, 2013). Expressions of gender identity affirmation from others in the social environment range from correctly using one’s chosen name and pronouns to broader recognition of one’s chosen gender identity (Glynn et al., 2016; Reisner et al., 2016b; Russell et al., 2018). Indeed, past work has shown that having one’s gender identity affirmed is associated with lower depressive symptomatology (e.g., Glynn et al., 2016; Nuttbrock et al., 2012; Russell et al., 2018) and greater self-esteem (Glynn et al., 2016) among TGNC people.

The process by which experiences of gender identity affirmation might improve well-being has, however, not been explored in past work. Here, we suggest that self-concept clarity (Campbell, 1990) might act as an important mechanism. Clarity around one’s self-concept—the sense that one knows confidently who one is—has been argued to be an important foundation for adaptive functioning. This idea is supported by the consistent association between self-concept clarity and markers of psychological health, such as self-esteem, and reduced neuroticism, anxiety and depression (Bigler et al., 2001; Campbell, 1990; Campbell et al., 1996, 2003). It seems plausible that having one’s gender identity affirmed by others (and thereby having the internal self-concept verified) should lead to a greater sense of coherence and self-concept clarity (Barreto & Ellemers, 2003; Campbell, 1990; Swann, 1983). Consistent with this broad idea, correlational research among bisexual individuals has revealed that the experience of identity denial is (1) separable from the experience of discrimination, and (2) associated with reduced self-concept clarity and reduced mental health and self-esteem (Garr-Schultz & Gardner, 2019). Other correlational studies show that individual self-concept clarity mediates the effects of collective uncertainty on individual well-being (Usborne & Taylor, 2010). Finally, experimental studies further show that individual self-affirmation increases momentary self-concept clarity (e.g., Wakslak & Trope, 2009) and conversely that experiences of interpersonal rejection undermine this, especially among those who are sensitive to rejection (Ayduk et al., 2009). Along these lines, we suggest that having others confirm and actively recognize a core element of the self might reinforce the confidence and coherence TGNC experience around their self-concept, something that should be beneficial for their well-being.

### The Current Study

The aim of the current study was to model the associations among perceived discrimination, transgender identification, gender identity affirmation and well-being among TGNC people. Based upon theory on stigma-related stress and resilience among TGNC people (e.g., Hendricks & Testa, 2012; Sevelius, 2013), we hypothesized that perceived discrimination would be associated with poorer well-being, whereas transgender identification and gender identity affirmation would be associated with greater well-being. Furthermore, based on previous work suggesting that group identification can buffer minority individuals against the negative experience of discrimination (e.g., Hansen & Sassenberg, 2006; Mossakowski, 2003), we hypothesized that transgender identification would moderate the association between perceived discrimination and well-being. Specifically, we expected that the negative association between perceived discrimination and well-being would be attenuated among those TGNC people who were more highly identified with the transgender community. Finally, and in a more exploratory way, we tested whether gender identity affirmation would similarly moderate associations between perceived discrimination and well-being and whether gender identity affirmation and well-being might be indirectly related via the positive consequences of affirmation for individual self-concept clarity.

## Method

### Participants and Procedure

Participants were 105 self-identified TGNC individuals (i.e., individuals who do not identify with the sex assigned to them at birth). In addition to confirming their self-identification as TGNC as an eligibility check for the study, participants were asked to describe their gender identity using an open-ended response option. These responses were coded as transgender versus gender non-conforming by the researchers for descriptive purposes, with 42% of the sample identifying as transgender male, 39% transgender female, and 19% gender non-conforming (e.g., non-binary, gender fluid, gender queer). The sample was predominantly White (91% identified as White/Caucasian) and on average middle aged (*M* = 40.66, *SD* = 17.05). Only a third of the sample earned £40,000 per year or more (with 40% earning less than £20,000). In reporting when they started feeling that they did not identify with the sex they were assigned at birth, 69% indicated “less than a year ago,” 29% indicated “a few years ago,” and 2% indicated “as long as [they] can remember.” A majority of participants (64%) reported having previously used or currently using any gender identity services (public or private). Participants were recruited through online forums and support groups in the UK and North America used by transgender individuals to get in touch with and share experiences with other members of the transgender community (with 87% of participants from the UK and 13% from North America). They followed a link to an online survey (hosted by Qualtrics). Participation was voluntary (no remuneration provided).

### Measures

#### Perceived Discrimination

Three items measured individuals’ experiences with discrimination as a transgender person (adapted from Schmitt et al., 2002). Participants were prompted to respond to the following items thinking about their “experiences in society as a transgender individual”: “I have personally been a victim of discrimination because of my gender identity,” “I consider myself a person who has been deprived of opportunities because of my gender identity,” and “I feel like I personally have been a victim of society because of my gender identity.” Items were rated on a seven-point scale (1 *strongly disagree–*7 *strongly agree*) and were internally consistent (*α* = 0.89).

#### Transgender Identification

Individuals’ identification as a transgender person was measured using items adapted from Leach et al. (2008). Items assessed two identity components: satisfaction/pride (three items, e.g., “I am glad to be transgender”) and solidarity (three items, e.g., “I feel solidarity with other transgender people”). Items were rated on a seven-point scale (1 *strongly disagree–*7 *strongly agree*) and were internally consistent (α ≥ 0.77 for each component).

#### Gender Identity Affirmation

Four items assessed individuals’ experiences of gender identity affirmation: “People generally refer to me using my chosen pronouns,” “People tend to refer to me by using my chosen name,” “I feel that people generally acknowledge my preferred gender identity,” “I often feel that other people do not see me the way I want to be seen (reverse scored).” Items were rated on a seven-point scale (1 *strongly disagree–*7 *strongly agree*) and were internally consistent (*α* = 0.88).

#### Well-Being (Self-esteem, Life Satisfaction)

Well-being was measured using scales of personal self-esteem (general self-esteem; six items, e.g., “On the whole, I am satisfied with myself;” Rosenberg, 1965; and appearance-based esteem; three items, e.g., “I feel satisfied with the way my body looks;” Heatherton & Polivy, 1991) and life satisfaction (five items, e.g., “I am satisfied with my life;” Diener et al., 1985). General personal self-esteem items were measured on a five-point scale (1 *strongly disagree–*5 *strongly agree*; *α* = 0.84), appearance self-esteem was on a five-point scale (1 *not at all–*5 *extremely*; *α* = 0.86), and life satisfaction was on a seven-point scale (1 *strongly disagree–*7 *strongly agree*; *α* = 0.86).

#### Self-Concept Clarity

Three items assessed individuals’ sense of having a clear sense of self (Campbell et al., 1996): “In general, I have a clear sense of who I am and what I want,” “Sometimes I feel that I am not really the person that I appear to be (reverse scored),” “My beliefs about myself often conflict with one another (reverse scored).” Items were rated on a five-point scale (1 *strongly disagree–*5 *strongly agree*) and were internally consistent (*α* = 0.73).

### Analyses

We conducted analyses using structural equation modeling (SEM) in EQS software. All constructs were specified as latent factors (perceived discrimination, gender identity affirmation, and self-concept clarity using their respective measurement items as indicators, group identification and well-being using composites of identity satisfaction and solidarity, and general self-esteem, appearance esteem, and life satisfaction as indicators, respectively) enabling unbiased estimates of structural parameters without an overly complex measurement model. The discrimination–identification interaction term was also specified as a latent factor (as was the exploratory discrimination–affirmation interaction term), with two indicator variables each representing cross-product terms created from mean centered main effect variable indicators using a matched pairs strategy (Marsh et al., 2004). We tested simple slopes in SEM following guidelines from Aiken and West (1991). Note that latent factors representing main effects (discrimination, identification) were specified using mean centered indicators (as was gender identity affirmation when used for specifying an interaction term). All manifest indicators in each model were predicted by their respective latent factors at *p* ≤ 0.01. To maximize use of data and address the presence of multivariate non-normality, we utilized robust full information maximum likelihood estimation (FIML; Bentler, 2006; Satorra & Bentler, 1990; Yuan & Bentler, 2000; Yuan et al., 2004). FIML allows retention of data for all cases by estimating each parameter based upon all available data, recommended over other approaches to missing data (e.g., mean substitution, list-wise deletion; Enders & Bandalos, 2001; Johnson & Young, 2011).

In primary analyses, we tested: (1) whether experiences of discrimination were associated with well-being and (2) whether identity-based resilience factors played a unique and important role in explaining TGNC individuals’ well-being over and above experiences with discrimination. This was done by specifying a model in which well-being was regressed on perceived discrimination, alongside transgender identification and gender identity affirmation. Furthermore, we tested a discrimination–identification interaction term (as a predictor of well-being). In exploratory follow-up analyses, we specified a model paralleling our primary analyses but with a discrimination–affirmation interaction term (examined both in place of and alongside the hypothesized discrimination–identification term). Finally, in a separate set of analyses we examined an indirect association between gender identity affirmation and well-being via self-concept clarity.

## Results

Summary statistics and bivariate correlations are presented in Table 1. In exploratory analyses, we compared means on all constructs in the study between those who self-identified as transgender (*n* = 85) and those who self-identified as gender non-conforming (*n* = 20). These analyses revealed no statistically significant differences between groups, so we proceeded to test the full model including all participants in the sample.

**Table 1:** Means, standard deviations, and bivariate correlations among key variables

Variable	*M*	*SD*	1	2	3	4	5	6
1. Perceived discrimination	4.17^a^	1.84	–					
2. Transgender identification	4.63^a^	1.21	.09	–				
3. Gender identity affirmation	4.93^a^	1.53	− .19^+^	− .16	–			
4. Self-esteem (general)	3.48^b^	0.98	− .31**	.13	.38***	–		
5. Self-esteem (appearance)	2.44^b^	1.04	− .27*	.07	.32**	.70***	–	
6. Life satisfaction	3.36^a^	1.46	− .28*	.24*	.25*	.67***	.68***	–
7. Self-concept clarity	3.08^b^	1.10	− .29**	-.04	.44***	.58***	.58***	.51***

^a^1–7 scale, ^b^1–5 scale. ***p ≤ .001, **p ≤ .01, *p ≤ .05, ^+^*p* ≤ .10

### The Importance of Identity-Based Resilience Factors to TGNC People’s Well-being

**Fig. 1:**
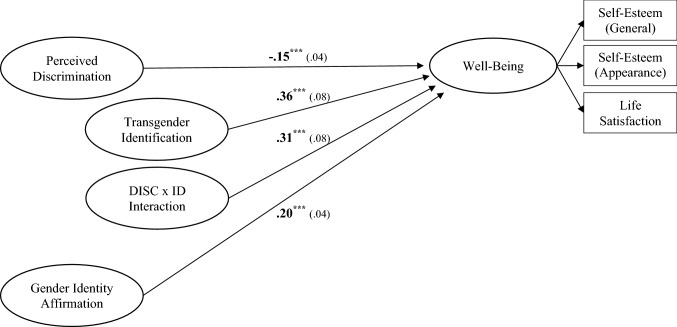
Results of SEM regression analyses with unstandardized path coefficients (standard errors), showing identity-based resilience factors predicting TGNC individuals’ well-being (over and above their experiences with discrimination). Factor loadings are omitted for simplicity though all lambdas were associated with their respective latent factors at *p* < .01. Probing the discrimination–identification interaction (DISC x ID) revealed that stronger transgender identification (at + 1 *SD*) buffered the link between discrimination and well-being (*B* =  − .06, *p* = .62). For individuals lower in identification (at − 1 *SD*), experiences of discrimination were linked to lower well-being (*B* =  − 1.00, *p* < .001). An alternative model tested but found no evidence that gender affirmation moderates the link between discrimination and well-being. ***p ≤ .001

Primary analyses (shown in Fig. 1) revealed that the hypothesized model fit the data well, YB *χ*^2^(73) = 78.34, *p* = 0.31, CFI > 0.99, RMSEA < 0.001 [0.00, 0.05]. All parameters were significant as hypothesized, and the model accounted for a substantial portion of variance in well-being (*R*^2^ = 0.58). As expected, individuals who experienced more discrimination had lower levels of well-being (*B* =  − 0.15, *p* < 0.001), though this was qualified by a discrimination–identification interaction (*B* = 0.31, *p* < 0.001), such that greater identification buffered the discrimination-well-being link (there was also a main effect of identification, *B* = 0.36, *p* < 0.001). Specifically, while discrimination was linked to lower well-being among individuals lower in transgender identification (-1 *SD*; *B* =  − 1.00, *p* < 0.001), for individuals higher in transgender identification (+ 1 *SD*), experiences of discrimination were not significantly associated with well-being (*B* =  − 0.06, *p* = 0.62). Additionally, as expected, individuals who more frequently experienced gender identity affirmation had greater levels of well-being (*B* = 0.20, *p* < 0.001).

To further probe these processes, we tested two modified models. The first was tested by specifying a model where gender identity affirmation was removed (otherwise identical to the hypothesized model). While this model still demonstrated good fit to the data, there was a clear decrease in its capacity to explain TGNC people’s well-being (*R*^2^ = 0.49). Indeed, the increased variance explained by accounting for gender identity affirmation represented a rather sizable effect: local effect size, Cohen’s *f* ^2^ = 0.22 (Cohen, 1988). The second modified model examined the strength of relationships between gender affirmation, discrimination, and identification. It mirrored the hypothesized model, but each of these latent factors was allowed to correlate. Results demonstrated that the correlations between them were all modest and often nonsignificant (discrimination–identification, *r* = 0.14, *p* = 0.18; affirmation–discrimination, *r* =  − 0.23, *p* = 0.02; affirmation–identification, *r* =  − 0.16, *p* = 0.08). These results further indicated the conceptually distinct and unique role of gender identity affirmation in TGNC people’s well-being.

### Exploratory Follow-Up Analyses

#### Gender Identity Affirmation as a Moderator

In a model paralleling the one used in primary analyses but with a discrimination–affirmation interaction term, results showed that while discrimination, transgender identification, and gender identity affirmation remained significant predictors of well-being in their own right (as in primary analyses), gender identity affirmation did not moderate the discrimination-well-being link (discrimination–affirmation interaction term: *B* =  − 0.06, *p* = 0.36). Overall model fit was also noticeably worse, e.g., YB *χ*^2^(73) = 117.24, *p* = 0.001). For thoroughness, we also tested a model with both gender identity affirmation and transgender identification as moderators simultaneously. Again, gender identity affirmation did not moderate the discrimination-well-being link, yet all other paths, as hypothesized, remained significant.

#### Indirect Effect Through Self-Concept Clarity

**Fig. 2:**
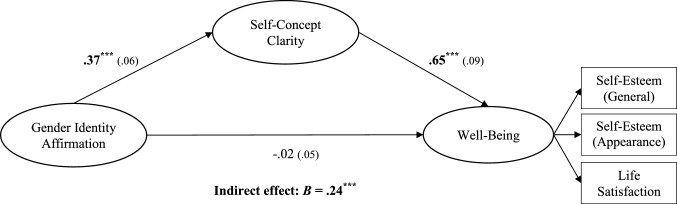
Results of follow-up SEM regression analyses of the indirect effect of gender identity affirmation on well-being through self-concept clarity with unstandardized path coefficients (standard errors). ***p ≤ .001

Finally, we tested whether self-concept clarity accounted for the link between gender affirmation and well-being (shown in Fig. 2). Results supported this hypothesis (indirect effect: *B* = 0.24, *p* < 0.001; direct effect, *B* =  − 0.02, *p* = 0.70). Thus, TGNC people who experienced greater gender identity affirmation had a greater sense of clarity about who they are as individuals (*B* = 0.37, *p* < 0.001) and this sense of clarity was in turn associated with greater well-being (*B* = 0.65, *p* < 0.001).

## Discussion

This study tested links between discrimination, identity, and well-being in TGNC individuals. The results replicated the association between perceived discrimination and well-being obtained with other types of stigmatized groups and showed that transgender identification potentially functioned as a protective factor for TGNC individuals. Specifically, while individuals who identified weakly as transgender reported lower well-being as a function of perceived discrimination, those who identified strongly as transgender did not show evidence of this deleterious association. This is consistent with research highlighting how a strong sense of identity is a source of resilience, even among members of socially stigmatized groups, for whom group membership is often a basis of devaluation (Leach et al., 2010).

In addition, this study was among the first to test the role of gender identity affirmation as an additional resilience factor for TGNC people’s well-being. Identity affirmation consists of treatment by others that validates and affirms one’s identity, rather than neglecting or denying it, and is particularly important when there is a potential for discrepancy between internal and external views of the self. This is often the case when identities challenge binary and fixed identity models, such as when migrants identify with the host country and feel their credentials for claiming these identities are questioned (Barreto et al., 2003). We examined this among TGNC people and found that identity affirmation was clearly distinct from transgender identification and experiences of discrimination and that identity affirmation was associated with well-being over and above the interactive effects of transgender identification and discrimination. Thus, overall, while discrimination was linked to lower well-being, TGNC people’s well-being was also related to key resilience factors, including transgender identification and experiences of having their gender identity recognized and affirmed.

The importance of gender identity affirmation for TGNC individuals is hard to overestimate. Having gender identity recognized and affirmed by others is a critical aspect of gender identity transition (i.e., it is a core element of ‘social transition’; Collazo et al., 2013). Social transition is sometimes the only transition in which TGNC people engage, or the first step taken by individuals who aspire to transition in other ways as well. Our findings show that not having one’s identity affirmed is clearly associated with poorer well-being.

This research also sheds light on *why* gender identity affirmation plays such a crucial role in well-being (i.e., by increasing self-concept clarity). As others have argued, social identities are not held in a social vacuum, and one important function they fulfill is that of placing oneself within the social world in relation to others (e.g., Deaux & Ethier, 1998). If identities are questioned, or neglected, this undermines their relational function. The sense of self-doubt that follows from such experiences is likely to create uncertainty around the self (Ayduk et al., 2009) and through this be damaging to well-being (Barreto et al., 2010; Garr-Schultz & Gardner, 2021; Usborne & Taylor, 2010). The process through which TGNC people develop and internalize a sense of clarity about who they are as individuals may be one that works, in part, from the outside-in. When others treat TGNC individuals in ways that offer recognition and validation of their gender identity, they may be more likely to embody a strong sense of clarity about who they are as individuals themselves.

Though these findings are important, the cross-sectional methodology limits our ability to draw causal inferences for the paths examined in this research. It is possible that other confounding factors that we could not include in our models (primarily in order to preserve statistical power) account for some portion of observed associations. On the topic of statistical power, this study is also limited by the relatively small sample. Importantly, this sample size was determined not by a priori power analysis, but rather practicalities of recruitment for the current study. Given the proportion of latent factors to manifest variables in the hypothesized model and the minimum absolute effect detected among hypothesized structural parameters (*r* = 0.32), the minimum sample size to detect specified effects was indicated as 129, while the minimum sample size for the specified model structure was indicated as 232 (α = 0.05, 1 − *β* = 0.80; Soper, 2020). Other rule-of-thumb recommendations for adequate power in SEM would suggest a minimum sample size of 140 (i.e., 10 participants per indicator variable; Nunnally, 1967), but in either case, our sample size fell somewhat short of the number of participants required to achieve power of 0.80. Because of this, results of the current models should be interpreted with caution. However, it must be acknowledged that recruiting large samples can be a significant challenge when conducting research with TGNC populations, potentially limiting the types of health research questions addressed (with studies achieving larger samples often focusing on substance use, sexual health or mental health problems; Reisner et al., 2016a; Reisner et al., 2016b; Reisner et al., 2016c).

In future research, longitudinal studies with TGNC people (which may capitalize on within-person analyses to improve statistical power) will be essential to uncover how these processes unfold over time and throughout gender identity transition. Future studies might also aim to replicate these findings with different measures that have been developed with careful attention to psychometric properties (as our measures of perceived discrimination and gender identity affirmation, for example, were somewhat ad hoc and adapted for use in the current research). For example, a recent systematic review of psychometric properties of measures of perceived discrimination for transgender people (Morrison et al., 2018) identified four ‘gold-standard’ scales/subscales that could be used in future work on this topic.

Despite the limitations of this study, our findings pave the way for new and exciting research directions. Qualitative research might contribute to shed further light on other possible mechanisms through which identity affirmation might affect well-being. Moreover, future research might further probe what role gender identity affirmation (or lack thereof) might play in shaping other aspects of gender identity transition. By illustrating how integral social and identity-related processes are to the well-being of TGNC people, this research makes an important contribution to the understanding of the dynamics surrounding TGNC well-being and social gender identity transition.

## Data Availability

The materials and anonymized data for this study are available upon request to the corresponding author.

## Code availability

The code for analyses conducted in this work are available upon request to the corresponding author.
